# Isolation and characterization of the phytopathogenic fungus *Ilyonectria liriodendri* from persimmon as a new susceptible host

**DOI:** 10.1371/journal.pone.0339616

**Published:** 2025-12-26

**Authors:** Nikolett Molnár, Dóra Szabó, Adrienn Geiger, József Geml, Kálmán Zoltán Váczy, Zoltán Karácsony

**Affiliations:** 1 Food and Wine Research Institute, Eszterházy Károly Catholic University, Eger, Hungary; 2 ELKH-EKKE Lendület Environmental Microbiome Research Group, Eszterházy Károly Catholic University, Eger, Hungary; Benemérita Universidad Autónoma de Puebla: Benemerita Universidad Autonoma de Puebla, MEXICO

## Abstract

Several members of the fungal genus *Ilyonectria* primarily infect plants through the roots and basal stem, causing ‘black foot’ diseases, predominantly in woody plants such as grapevine (*Vitis* spp.) and walnut (*Juglans regia*). In 2021, four *Ilyonectria liriodendri* isolates were cultured from the necrotized roots of *Diospyros virginiana* plants in Eger, Hungary. The isolates were identified by sequencing the ITS, β-tubulin, and partial histone H3 genes. The obtained sequences were used for phylogenetic analysis through multiple sequence alignment and the construction of a Maximum Likelihood tree, which revealed that all four isolates belonged to the species *Ilyonectria liriodendri*. The macro- and micromorphological variations, as well as the differences in exoenzyme production of the isolates suggested that they represent a somewhat diverse set of the same taxon. To prove their association with the symptoms observed in the host plants, the roots of one-year-old *D. virginiana* plants were artificially infected with conidial suspensions of the isolates according to Koch’s postulates. After 90 days of incubation in a greenhouse, 16 out of 20 inoculated plants showed necrosis in the taproots, while mock-inoculated plants remained symptomless. Necroses developed in the roots of the infected plants, and the inoculated fungi were reisolated, reinforcing their pathogenicity against *D. virginiana*. To the best of our knowledge, this is the first report of *I. liriodendri* causing disease in persimmon.

## Introduction

The genus *Ilyonectria* belongs to the ascomycetous family Nectriaceae and was described in 2014 [1], with 16 known species to this date (https://www.mycobank.org/). While *Ilyonectria* species are mostly known as phytopathogens, they can grow as saprobes as well as endophytes [2,3]. Most of these species are considered opportunistic soil-borne pathogens of various plants, infecting the roots and the base of the hosts. These pathogens damage the xylem, resulting in “black root rot” and “black foot disease” syndromes [4]. They cause diseases mostly on woody plants like grapevine [5], apple [6], plum, tulip tree [7], loquat [8], olive trees [9], and blackberry [10]. The most widely studied syndrome caused by *Ilyonectria* spp. is the “black foot disease” of grapevine [7,11,12], a member of the so-called grapevine trunk diseases group [13]. Their common symptom is the necrosis of the infected xylem tissues, appearing as black discolorations. The damage to the vascular tissues, probably with the additional effects of fungal phytotoxins [14], results in the decline and the eventual death of the host. While there is no estimation on the economic loss caused by grapevine black foot diseases, both the syndrome and pathogens show a high incidence worldwide [11,15–17].

Persimmon species (*Diospyros* spp.) are also susceptible to vascular infections, causing host decline and significant yield losses [18]. The only bacterial pathogen causing persimmon dieback is *Pseudomonas syringae* [19]. Most of the causal agents are fungal species which also associated with different grapevine trunk disease syndromes. These fungi include *Neofusicoccum* and *Diplodia* species (botryosphaeria dieback), *Eutypa lata* (eutypiosis), *Phaeoacremonium* species (esca disease), *Diaporthe* species (phomopsis disease), as reported by Moyo et al. [20]. The only exception is the ascomycetous *Colletotrichum horii* [21] with no known connection with grapevine trunk diseases. In this context, the lack of fungal species associated with grapevine black foot disease in persimmon is conspicuous.

In 2021, declining young *Diospyros virginiana* plants were investigated in Hungary, showing necrotic spots in their taproots. The fungal species *Ilyonectria liriodendri* was isolated from most of the root samples according to the morphological characteristics and the analysis of ITS, β-tubulin, and partial histone H3 gene sequences of the isolates. To the best of our knowledge, this is the first report of *I. liriodendri* pathogen causing infection in persimmon.

## Materials and methods

### Isolation of fungal strains

**Fig 1:**
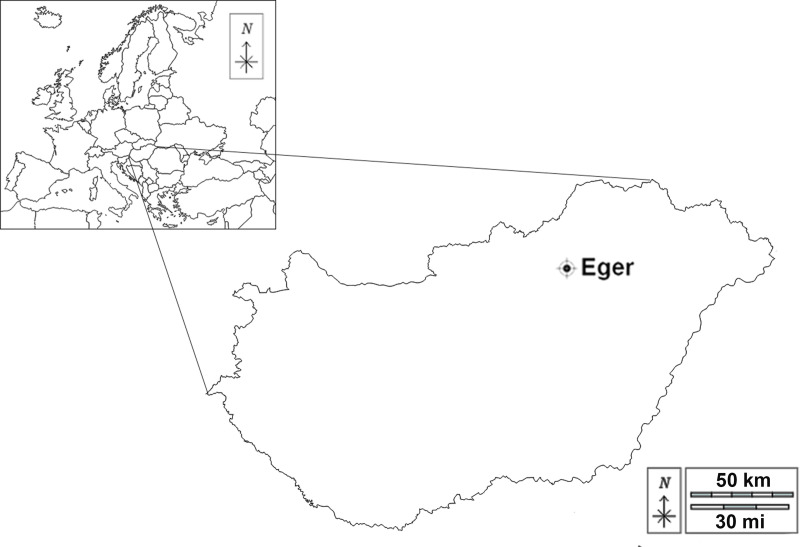
The origin of fungal strains. Map showing the location of the fungal isolates obtained in Hungary. The figure was created using online resources (https://d-maps.com/m/europa/hungary/hongrie/hongrie06.gif; https://d-maps.com/m/europa/europemax/europemax10.gif).

Xylem samples were collected from the taproots of 15 declining *D. virginiana* plants in Eger city (Northeastern Hungary, Fig 1), in 2021 from a hobby grower. Five thin discs were cut from each root. The isolation of fungi was carried out as described previously [22]. After the bark tissues were removed, the discs were surface-sterilized in 1% chloramine B solution for 5 min. The sterilized tissues were rinsed in sterile distilled water and dried. Then each sterilized discs were cut into five pieces and placed on potato dextrose agar plates (PDA, Merck KGaA, Darmstadt, Germany). The plates were incubated at room temperature (21 ± 2°C) in the dark for two weeks. Cultures were checked daily, and emerging mycelia were subcultured to new PDA plates to acquire pure cultures for further morphological and molecular identification. A total of 173 fungi were isolated, including 11 *Ilyonectria*-like isolates (from 11 different plants). The *Ilyonectria*-like isolates could be divided into four morphological groups. One representative isolate of each morphological group was subjected to detailed examination.

### Molecular identification and phylogenetic analyses of fungal isolates

Mycelia from one-week-old PDA cultures were scraped and placed in a microcentrifuge tube. Fungal biomass was lyophilized and ground by the use of sterilized stainless steel bead and Tissuelyzer LT device (Qiagen, Hilden, Germany) at 50 Hz, for 5 min. DNA was extracted by DNeasy Plant Mini Kit (Qiagen, Hilden, Germany) according to the manufacturer’s instructions. Polymerase chain reactions (PCRs) were performed to amplify the partial H3 histone gene using CYLH3F (5’-AGGTCCACTGGTGGCAAG-3’) and CYLH3R (5’-AGCTGGATG TCCTTGGACTG-3’) primers; the ITS region using ITS4 (5’-TCCTCCGCTTATTGATATGC-3’) and ITS1F (5’-CTTCGTCATTTAGAGGAAGTAA-3’) primers; and the β-tubulin gene using BT2a (5’-GGTAACCAAATCGGTGCTGCTTTC-3’) and Bt2b (5’-ACCCTCAGTGTAGTGACCCTTCGC-3’) primers as described previously [23]. PCR conditions were 94 °C for 5 min, followed by 35 cycles at 94 °C for 30 s, 52 °C for 30 s and 72 °C for 80 s, and a final elongation at 72 °C for 10 min [24]. The PCR products were visualised on 1% agarose gel. Amplicons were sequenced (BaseClear B.V. -Netherlands) and compared to GenBank using BLAST (www.ncbi.nlm.nih.gov) [25].

For the accurate identification of the examined isolates, the partial H3 histone, the ITS (internal transcribed spacer), and the β-tubulin genes were sequenced. The three previously mentioned loci of 61 *Ilyonectria*, *Dactylonectria* and *Neonectria* strains were collected from the NCBI nucleotide database (https://www.ncbi.nlm.nih.gov/nucleotide/) and used as a reference for the phylogenetic tree construction. Sequences were analyzed on the Phylogeny.fr platform [26] and subsequently aligned with MUSCLE (v3.8.31) [27]. The removal of ambiguous regions was done by Gblocks (v0.91b) using default parameters [28]. A phylogenetic tree was reconstructed according to the maximum likelihood method using the PhyML program (v3.1/3.0 aLRT) [29,30]. Tree visualization was done by TreeDyn (v198.3) [31]. All sequences used in the phylogenetic analysis are listed in Table 1. Identification of the fungal isolates was further verified by species-specific PCR reactions designed to detect *I. liriodendri*, using the primers Cyli F1 (5′-CTC CTC TTC AAC GAT CCG ACG TGC C-3′) and Cyli R1 (5′-GGG GCA GAG CAG ATT TCG-3′), producing a ~ 200 bp amplicon [6].

**Table 1:** Sequences used in the phylogenetic analysis. List of fungal strains and GenBank (https://www.ncbi.nlm.nih.gov/genbank/) sequence accession numbers. Strains described in this study are underlined.

	Strain ID	ITS sequence accession	H3 sequence accession	BTUBULIN sequence accession
Ily1	Ily1	PV931980	PP680573.1	PV943738
Ily3	Ily3	PV931981	PP680574.1	PV943739
Ily4	Ily4	PV931982	PP680575.1	PV943740
Ily5	Ily5	PV931983	PP680576.1	PV943741
Dactylonectria_alcacerensis_1	BV-1240	MK602786	MK579235	MK602801
Dactylonectria_alcacerensis_2	Cy133	JF735331	JF735628	JF735459
Dactylonectria_alcacerensis_3	129087	JF735333	JF735630	AM419111
Dactylonectria_estremocensis	CBS 112613	JF735320	JF735617	JF735448
Dactylonectria_macrodidyma_1	EFA446	MF440371	MF471474	MF797794
Dactylonectria_macrodidyma_2	CBS 112601	AY677284	JF735644	AY677229
Dactylonectria_macrodidyma_3	CBS 112615	AY677290	JF735647	AY677233
Dactylonectria_macrodidyma_4	CBS 112594	AY677282	JF735643	AY677231
Dactylonectria_macrodidyma_5	CBS 112603	AY677285	JF735645	JF735469
Dactylonectria_macrodidyma_6	CBS 112605	AY677287	JF735646	AY677230
Dactylonectria_macrodidyma_7	CBS 20709	JX231163	JX231147	JX231115
Dactylonectria_novozelandica_1	KARE2037	MK400310	MK409912	MK409877
Dactylonectria_peuciseptata_1	CBS 100819	EF607090	JF735582	EF607067
Dactylonectria_peuciseptata_2	CBS 120171	EF607089	JF735587	EF607066
Dactylonectria_torresensis_1	CBS_129086	NR121500	JF735681	JF735492
Dactylonectria_torresensis_2	CBS 119.41	JF735349	JF735657	JF735478
Dactylonectria_torresensis_3	CBS 112598	JF735351	JF735662	JF735479
Dactylonectria_vitis	CBS 129082	JF735303	JF735580	JF735431
Ilyonectria_capensis_1	CBS_132815	NR152887	JX231135	JX231103
Ilyonectria_changbaiensis_4404	txid2590834	MF350464	MF350437	MF350410
Ilyonectria_communis_1512	txid2590833	MF350456	MF350429	MF350402
Ilyonectria_coprosmae	CBS 119606	JF735260	JF735505	JF735373
Ilyonectria_crassa_1	CBS 139.30	JF735275	JF735534	JF735393
Ilyonectria_crassa_2	CBS 158.31	JF735276	JF735535	JF735394
Ilyonectria_cyclaminicola	CBS 302.93	JF735304	JF735581	JF735432
Ilyonectria_cyclaminicola_1	CBS_302_93	NR121495	JF735581	JF735432
Ilyonectria_europaea_1	CBS 129078	JF735294	JF735567	JF735421
Ilyonectria_europaea_2	CBS 537.92	EF607079	JF735568	EF607064
Ilyonectria_ilicicola_1	Cy-FO-225	KY676884	KY676866	KY676878
Ilyonectria_leucospermi_1	CBS_132809	NR152889	JX231145	JX231113
Ilyonectria_liriodendri_1	CBS_110_81	NR119565	JF735507	DQ178170
Ilyonectria_liriodendri_2	CBS 117527	DQ178165	JF735509	DQ178172
Ilyonectria_liriodendri_3	CBS 117526	DQ178164	JF735508	DQ178171
Ilyonectria_liriodendri_4	MBAE5MY	MT711168	MT708551	MT748009
Ilyonectria_liriodendri_5	MBAE7MY	MT711169	MT732971	MT748010
Ilyonectria_liriodendri_6	MBAE10MY	MT711170	MT732972	MT748011
Ilyonectria_liriodendri_7	CBS 117640	DQ178166	JF735510	DQ178173
Ilyonectria_liriodendri_8	CBS 112596	AY677264	JF735511	AY677239
Ilyonectria_liriodendri_9	CBS 112607	AY677269	JF735512	AY677241
Ilyonectria_mors-panacis_1	CBS 306.35	JF735288	JF735557	JF735414
Ilyonectria_mors-panacis_2	CBS 124662	JF735290	JF735559	JF735416
Ilyonectria_protearum_1	CBS_132812	NR152890	JX231149	JX231117
Ilyonectria_palmarum	DiGeSA-HF7	HF937432	HF922621	HF922609
Ilyonectria_pseudodestructans_2	CBS 117824	JF735292	JF735419	JF735562
Ilyonectria_pseudodestructans_3	CBS 129081	AJ875330	JF735563	AM419091
Ilyonectria_qitaiheensis_H309	txid2590835	MF350472	MF350445	MF350418
Ilyonectria_robusta_1	CBS_308_35	NR157427	JF735518	JF735377
Ilyonectria_robusta_2	CBS 129084	JF735273	JF735532	JF735391
Ilyonectria_rufa_1	CBS 153.37	AY677271	JF735540	AY677251
Ilyonectria_rufa_2	CBS 640.77	JF735277	JF735542	JF735399
Ilyonectria_vredehoekensis_1	CBS_132807	NR152888	JX231139	JX231107
Ilyonectria_venezuelensis	CBS 102032	AM419059	JF735571	AY677255
Ilyonectria_zarorii_1	CPC_37835	MW114893	MW119259	MW119263
Neonectria_ditissima_1	CBS 226.31	JF735309	JF735594	DQ789869
Neonectria_ditissima_2	CBS 835.97	JF735310	JF735595	DQ789880
Neonectria_major	CBS 240.29	JF735308	JF735593	DQ789872
Neonectria_neomacrospora	CBS 118984	JF735311	JF735598	DQ789882
Neonectria_ramulariae_1	CBS 151.29	JF735313	JF735602	JF735438
Neonectria_ramulariae_2	CBS 182.36	JF735314	JF735603	JF735439

CBS, Westerdijk Fungal Biodiversity Institute, Ultrecht, The Netherlands (https://wi.knaw.nl/).

### Morphological observations

One cm-wide mycelial disks were cut from the edge of one-week-old colonies growing on PDA and inoculated in the center of 9 cm-wide PDA, malt extract agar (MEA, Merck KGaA, Darmstadt, Germany), oatmeal agar (OA, Merck KGaA, Darmstadt, Germany), or 2%m/v water agar (WA) plates. Cultures were incubated at 25°C in the dark, for 14 days. For microscopic observations, fungal biomass was scraped from colonies growing on PDA, mounted in a drop of distilled water, and covered with a coverslip. Microscopic examinations were done by Olympus BX53F2 (Olympus Corporation, Tokyo, Japan) microscope equipped with differential interference contrast (DIC) optics. Photographs were taken by DP47 camera controlled by CellSens Entry software (Olympus Corporation, Tokyo, Japan). Conidial dimensions were determined by image analysis, using a Burker chamber as a reference, by measuring 25 microconidia and 25 macroconidia for each examined strain.

### Examination of exoenzyme production

The activity of digestive enzymes of Ily1, Ily3, Ily4 and Ily5 strains was compared. Czapek dox media (2% w/v sucrose, 0.2% w/v NaNO_3_, 0.1% w/v K_2_HPO_4_, 0.05% w/v MgSO_4_, 0.05% w/v KCl, 0.001% w/v FeSO_4_, 2% w/v agar) supplemented with various substrates of the different enzymes were prepared. Carboxymethylcellulose (1% w/v) was used for cellulase, water-soluble starch (1% w/v) for amylase and ABTS (2mM) for laccase activity detection.

The *I. liriodendri* strains were inoculated onto the above media, as mycelial plugs of 3 mm diameter growing on PDA medium. The effects of the observed digestive enzymes were detected after 7 days of incubation at 25°C. Lugol’s staining was carried out for amylase and cellulase detection, followed by the measurement of the radius of halos developed around the colonies. The visualization of laccase activity was observed through the fungal colonies green-blue colorization. Laccase activity was quantified by calculating the decrease in mean pixel intensities of coloured colonies relative to uninoculated media. All the inoculations and measurements were done in triplicate for each strain.

### Softwares

Photographs and microscopic images were processed by Adobe Photoshop CS6 demo version and image analyses were done by Fiji [32]. Statistical comparisons were done by GraphPad Prism 5 software (GraphPad Software, San Diego California USA, www.graphpad.com) using One-way ANOVA with Tukey’s post-hoc test.

### Phytopathogenicity tests

One-year-old seed-grown plants of *D. virginiana* were used to evaluate the virulence of fungal isolates. The taproots were cut back to ~5 cm length, and surface sterilized by immersing in 70% v/v ethanol for 5 min. Injured plants were inoculated by incubating their bases for 30 min in conidial suspensions (10^6^ conidia/mL in distilled water). Mock inoculations were done by placing plants in distilled water. Inoculated plants were potted in a 1:1 mixture of perlite and commercial soil and were grown in a greenhouse for 90 days. Discs were cut 5–10 mm above the inoculation points, photographed and cut into five pieces. These pieces were surface-sterilized in sodium hypochlorite (4%m/v chlorine) for 2 min, then incubated in 70%v/v ethanol for 2 min, dried, and placed on PDA medium. Cultures were incubated at 25°C for a week, then the emerging mycelia were transferred to new PDA plates. Pure cultures were used for DNA extraction and PCRs. To verify the identity of the re-isolated fungi, the ITS, and partial H3 histone gene were amplified and sequenced. All the infections were carried out on five plants for each fungal isolate and mock inoculation.

## Results and discussion

### Isolation and identification of *Ilyonectria liriodendri* from symptomatic persimmon

**Fig 2:**
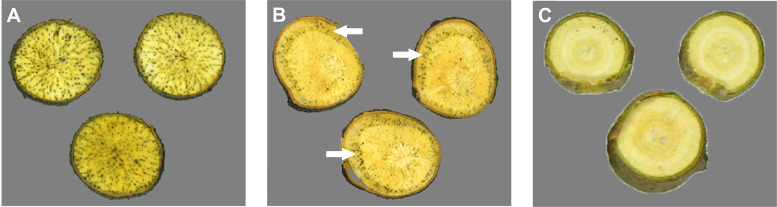
Symptoms on diseased persimmon plants. Representative photographs of taproot cross sections of *D. virginiana* plants with even **(A)** or circular **(B)** distribution of necroses, or with asymptomatic roots **(C)**.

The four examined declining *D. virginiana* plants showed black spots in the cross sections of the taproots (Fig 2). These spots are located in the tracheae resulting from tissue necrosis and/or vascular occlusion. The distribution of spots was even (Fig 2A), or circular (Fig 2B).

Four fungal strains (IDs: Ily1, Ily3, Ily4 and Ily5) isolated from symptomatic plants showed similar colony morphology as described for *I. liriodendri* [33], although slight differences were observed among the isolates. The abundant presence of 0–1 septate microconidia (average length between 6.08 and 9.17 µm depending on the isolate, S1 Table), and 1–3 septate macroconidia (average length between 11.86 and 35.23 µm depending on the isolate, S1 Table), as well as the lack of chlamydospores was observed in all examined strains. The above traits are also typical of *Ilyonectria* species [1]. Molecular identification of the isolates was done by multi-locus phylogenetic analysis using partial H3 histone, ITS, and β-tubulin gene sequences.

### Phylogenetic analyses

**Fig 3:**
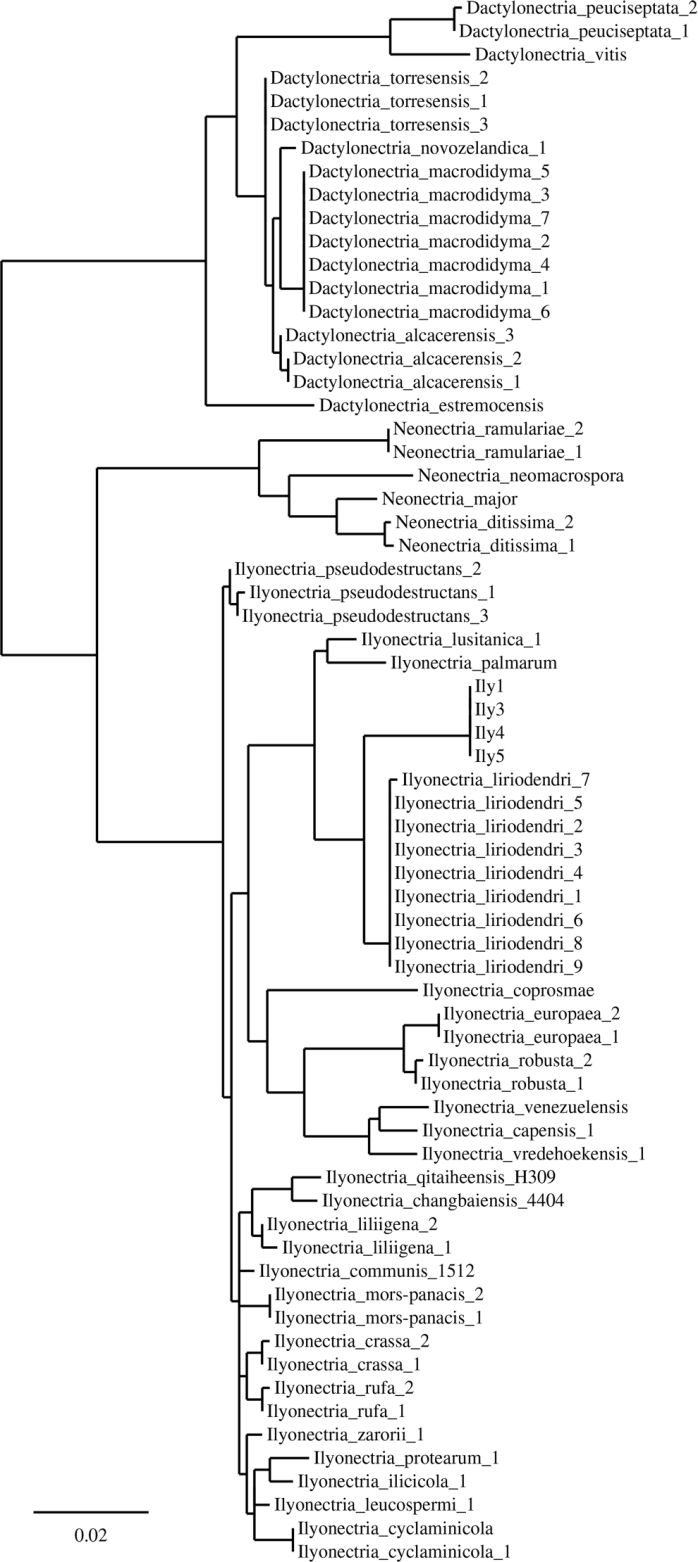
Phylogenetic analysis of fungal strains. Maximum Likelihood analysis of three genes (ITS + TUB2 + HIS) combined dataset in comparison with the four Ily isolates (Ily1, Ily3, Ily4 and Ily5)*.* Branches with a support value below 75% are collapsed.

The sequences of all four isolates showed the highest similarity to *I. liriodendri* strains based on the three previously mentioned loci and the constructed phylogenetic tree (Fig 3). Despite this similarity, the examined strains formed a separate sister clade with *I. liriodendri* reference strains. To verify that the examined isolates are *I.liriodendri*, species-specific PCR reactions were carried out. The reactions resulted in the expected ~200 bp length amplicon in case of all tested isolates, reinforcing that they belong to *I. liriodendri* (S1 and S2 Figs).

### Morphological characterization of *Ilyonectria liriodendri* isolates

**Fig 4:**
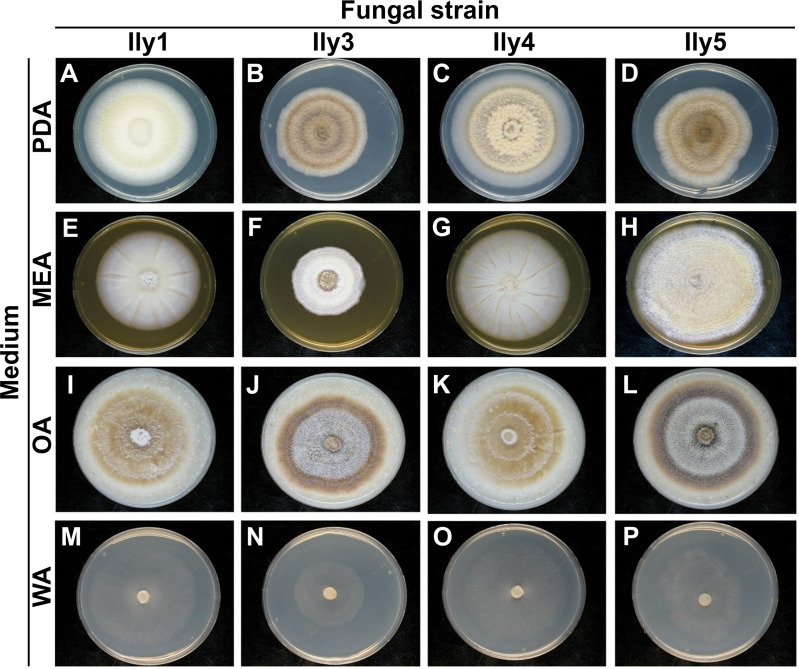
Macromorphological characterization of fungal colonies. Representative photographs *I. liriodendri* isolates (Ily1, Ily3, Ily4 and Ily5) grown for 14 days, at 25 °C, in the dark, on potato dextrose agar (PDA), malt extract agar (MEA), oatmeal agar (OA), and water agar (WA).

The morphological differences among the four examined isolates suggest that each of them represents a different strain. All isolates formed colonies with even margins on all tested media (Fig 4), but their coloration and relative growth differed. Strain Ily1 formed lemon-yellow colonies on PDA (Fig 4A), Ily4 was pale brown (Fig 4C), while Ily3 (Fig 4B) and Ily5 (Fig 4D) were dark brown on this medium. On MEA all strains showed a lighter coloration (Fig 4E–4H). Smooth, white colony surfaces were observed in the case of Ily1 and Ily4 (Fig 4E and 4G), while Ily3 and Ily5 colonies were both pale yellow and more fluffy (Fig 4F and 4H). Contrary to their coloration, the growth rates of Ily3 and Ily5 greatly differed on MEA medium (1.56 mm/day and 2.86 mm/day respectively, Fig 4F and 4H). Colonies grown on OA showed the similarity of Ily1 with Ily4 and Ily3 with Ily5 (Fig 4I–4L). Colonies of Ily1 and Ily4 were pale brown (Fig 4I and 4K), while Ily3 and Ily5 were dark brown (Fig 4J and 4L). On WA, all isolates formed thin mycelia without significant coloration (Fig 4M–4P), but the lower growth rate of Ily3 (1.24 mm/day) compared to the other strains (1.5–2.1 mm/day) was expressed again, as observed previously on MEA medium.

**Fig 5:**
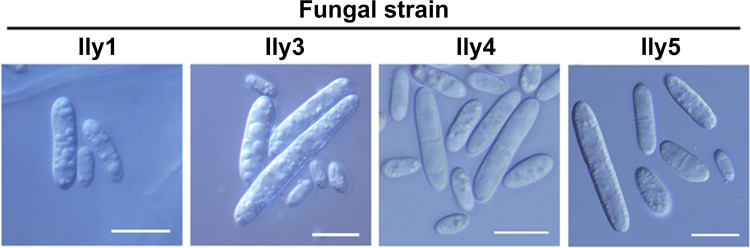
Asexual spores of fungal strains. Representative DIC photomicrographs of micro- and macroconidia of the examined *I. liriodendri* isolates (Ily1, Ily3, Ily4 and Ily5). Scalebars represent 10 µm.

Microscopic examination of the asexual spores of the *I. liriodendri* isolates (Fig 5) also revealed differences. Micro- and macroconidia were both observed in all strains.

**Fig 6:**
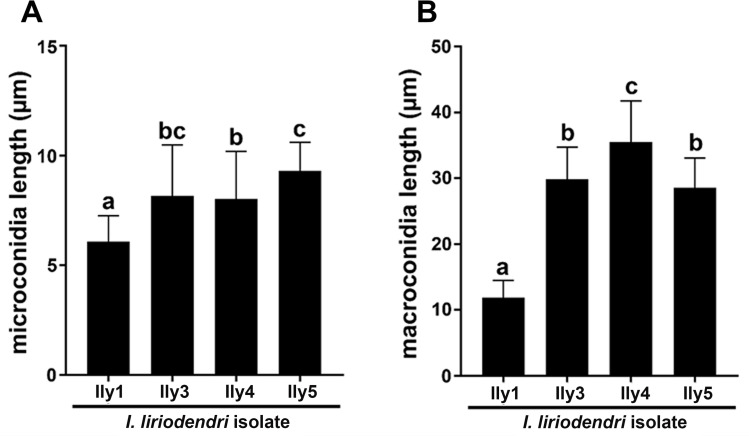
Characterization of fungal strains according to conidial dimension. Mean values and standard deviations of the length of microconidia **(A)** and macroconidia **(B)** of the examined *I. liriodendri* isolates (Ily1, Ily3, Ily4 and Ily5), grown on PDA medium, at 25 °C, for 14 days. Letters mark significantly (p < 0.05) differing datasets.

The characterization of conidial morphology (Fig 6 and S1 Table) further reinforced that the four *I. liriodendri* isolates are not clones, but represent a somewhat diverse set of strains. While Ily1 and Ily4 can be barely distinguished according to colony morphology (Fig 4), Ily1 produced significantly shorter microconidia (average length: 6.08 µm, Fig 6A) and macroconidia (average length: 11.86 µm, Fig 6B) compared to any other examined isolate (microconidia average length: 8.17–9.17 µm, macroconidia average length: 29.46–35.23 µm).

### Exoenzyme production of *I. liriodendri* isolates

**Fig 7:**
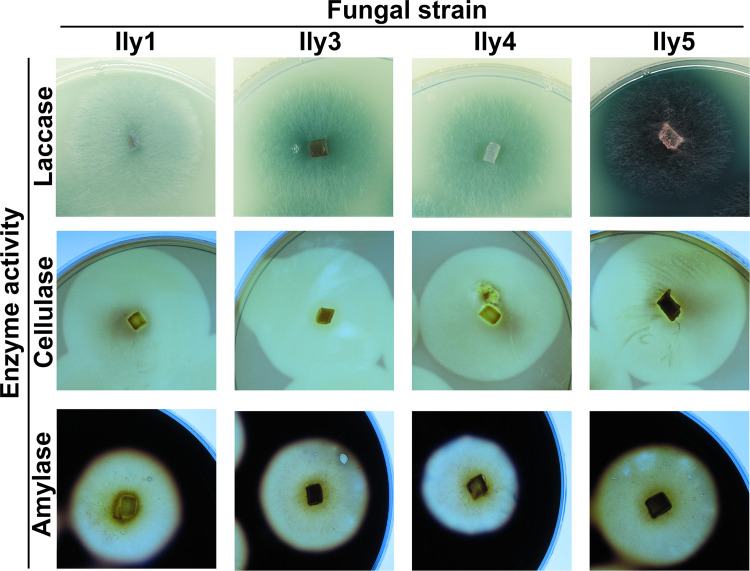
Exoenzyme production of fungal strains. Colonies of *I. liriodendri* Ily1, Ily3, Ily4 and Ily5 strains were grown on media indicating laccases **(A-D)**, cellulases **(E-H)** and amylases **(I-L)**. Photographs were taken after 7 days of incubation at 25°C.

**Fig 8:**
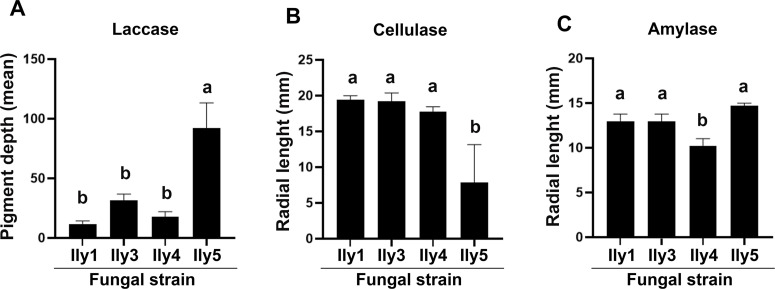
Quantification of fungal strains exoenzyme production. Laccase **(A)**, cellulase **(B)** and amylase **(C)** activities of *I. liriodendri* Ily1, Ily3, Ily4 and Ily5 strains. Different letters mark significantly differing (p < 0.05) datasets.

Secreted degradative enzymes are main pathogenicity factors of phytopathogenic fungi, and their activity usually correlates with virulence. Despite their crucial role in pathogenesis, they show high variance within species, making them suitable for strain characterization. Examination of the digestive exoenzymes revealed further differences among the four *I. liriodendri* strains (Figs 7 and 8 and S2 Table). In the case of laccase activity, the colonies of all strains showed different colorization strength, but the strongest colorization was observable at the Ily5 isolate, which was a significant difference according to the statistical analysis (Figs 7A–7D and 8A). All of the strains produced cellulases according to Lugol’s staining of carboxymethylcellulose-containing media, indicated by a clear zone around the colonies (Fig 7E–7H). The Ily 4 and Ily 5 strains, but especially the latter, showed significantly lower cellulase activity than the other strains (Fig 8B). On the starch-containing media, all four strains showed amylase activity (Fig 7I–7L), but in the case of Ily4 strain, this activity was significantly lower (Fig 8C).

### Pathogenicity tests

**Fig 9:**
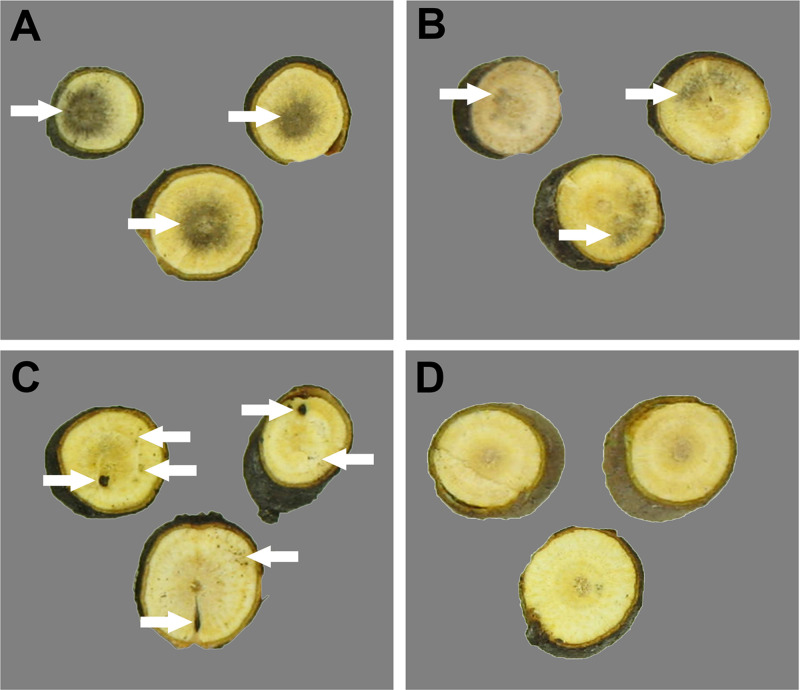
Symptoms caused by the fungal strains. Representative photographs of taproot cross sections of *D. virginiana* plants infected with *I. liriodendri* Ily1, Ily3, Ily4 and Ily5 isolates, or left uninoculated. Necroses with diffuse central **(A)**, diffuse lateral **(B)**, or spot-like **(C)** distribution were observed, while control plants remained symptomless **(D)**.

To verify the connection between the presence of *I. liriodendri* and the declining state of the hosts used for the isolation, pathogenicity tests were carried out on *D. virginiana* plants. After 90 days of incubation in a greenhouse, 16 out of the total 20 infected plants showed necrosis in the taproots, while mock-inoculated plants were symptomless (Fig 9 and Table 2). The incidence of symptoms was somewhat lower in the case of Ily3 and Ily5 isolates compared to the other strains. The lower pathogenicity of Ily3 and Ily5 cannot be clearly explained by the differences in exoenzyme activities (Fig 8). Both strains are efficient laccase (Fig 8A), and amylase (Fig 8C) producers. Although Ily3 showed low cellulase activity (Fig 8B), it can not explain why Ily5 is less virulent compared to Ily1 and Ily4. Possible connection between enzyme activities and virulence needs further studies.

**Table 2:** Symptom incidence and reisolation rate of fungal strains in pathogenicity assays.

	Isolate ID			
	Ily1	Ily3	Ily4	Ily5
symptom frequency	5/5	3/5	5/5	3/5
reisolation frequency	4/5	4/5	5/5	5/5

In most cases, the xylem necroses were diffuse, with central (Fig 9A) or lateral (Fig 9B) localization. Spotted necroses were detected only in three taproots (Fig 9C), out of the 16 symptomatic plants. The severity of root necroses showed high variances within a single fungal strain, hampering statistical comparisons. Contrary to the symptoms detected in artificially inoculated plants, necroses were observed in separate tracheae in the taproots of naturally infected persimmon (Fig 2). This difference is probably due to the combined effects of the excessive damage caused to the roots and the amount of the applied fungal inocula in the pathogenicity tests. The cutting of taproots and the application of high concentrations of fungal spores possibly resulted in the simultaneous infection of several tracheae, contrary to the infection of separate tracheae in naturally infected plants.

All tested strains can be reisolated frequently from the infected plants (Table 2), fulfilling the Koch postulates. The high number of causal agents, lack of specific symptoms, and the hidden nature of vascular fungal infections make it difficult to associate novel pathogens with these syndromes. The lack of available information on the pathogenicity of the extensively studied *I.* *liriodendri* pathogen on the worldwide cultivated persimmon host [34] raises the possibility that the observed disease is a result of specific conditions. The possible explanation could be the cultivation of the host outside its native range. *D. virginiana* is indigenous to the southeastern United States [35], while diseased plants were obtained in Hungary. Symptoms were observed in 2021, a year affected by drought and frequent heatwaves in the previous vintage [36]. These adverse climatic conditions may weaken the plants, making them more susceptible to diseases. In addition, in Hungary persimmon plants may encounter fungal strains with which it has not co-evolved. It is also notable that the origin of the diseased *D. virginiana* plants (Eger City) is the center of a significant wine region in Hungary [37] surrounded by numerous vineyards. No literature reports on the occurrence of grapevine black foot disease in the region. However, a previous DNA metabarcoding analysis suggests that *Ilyonectria* taxon is abundant in the local soil [38]. This study used the partial ITS sequence for identification, which is not suitable for species-level identification of fungi. However, it is easily possible that *I. liriodendri* was latently present in the soil*.* It was previously demonstrated that soil pathogen load is associated with host susceptibility to grapevine black foot disease [39]. In addition to soil, the grapevine host itself, as well as irrigation water and cutting tools, may also serve as a source of pathogen inocula [40].

## Conclusions

This study is the first report of the fungus *I. liriodendri* in persimmon based on morphological and molecular characterization of fungal isolates. In addition, pathogenicity tests led to the conclusion that *I. liriodendri* may contribute to the development of vascular fungal infection in the persimmon species, *D. virginiana*. The putative epidemiological relevance of this finding needs to be verified by examining the susceptibility of other members of the *Diospyros* genus to the infection. In addition, the distribution of the pathogenic trait against persimmon species also needs to be studied in geographically distant *I. liriodendri* populations.

The finding that *I. liriodendri*, a black foot disease pathogen, can infect *D. virginiana* has a potential impact on the persimmon industry. General practices of persimmon propagation and cultivation should be reconsidered, and new practices should be applied. Cutting back the taproot of persimmon rootstocks to enhance the growth of auxiliary roots is a general practice during propagation [41]. This provides a suitable entry point for the soil-borne black foot disease pathogens. Protection methods can be adapted from grapevine propagation, applying hot water [42], fungicides [43], or biocontrol agents [44] on roots. The mycorrhizal fungus *Glomus intraradices* would be a suitable candidate against black foot disease in persimmon since it can colonize and enhance the growth of *D. virginiana* [45], and has been proven to be a potent antagonist of black foot disease pathogens on grapevine [46].

## Supporting information

S1 Fig *I. liriodendri* species-specific PCR gel electrophoresis results. A clearly detectable amplicon at the expected size (~200 dp) in case of all four tested fungal isolates (Ily1, Ily3, Ily4, Ily5) was observed.
(TIF)

S2 Fig Original gel image for S1 Fig. (TIF)

S1 Table Raw data of micro- and macroconidia length of examined fungal strains in µm. (XLSX)

S2 Table Raw data of enzyme activities of examined fungal strains. (XLSX)
