# Supplementary figures and images for: Isolation and characterization of the phytopathogenic fungus Ilyonectria liriodendri from persimmon as a new susceptible host
Source: PLoS One. 2025 Dec 26;20(12):e0339616. doi: 10.1371/journal.pone.0339616 (PMC12742776; doi:10.1371/journal.pone.0339616)

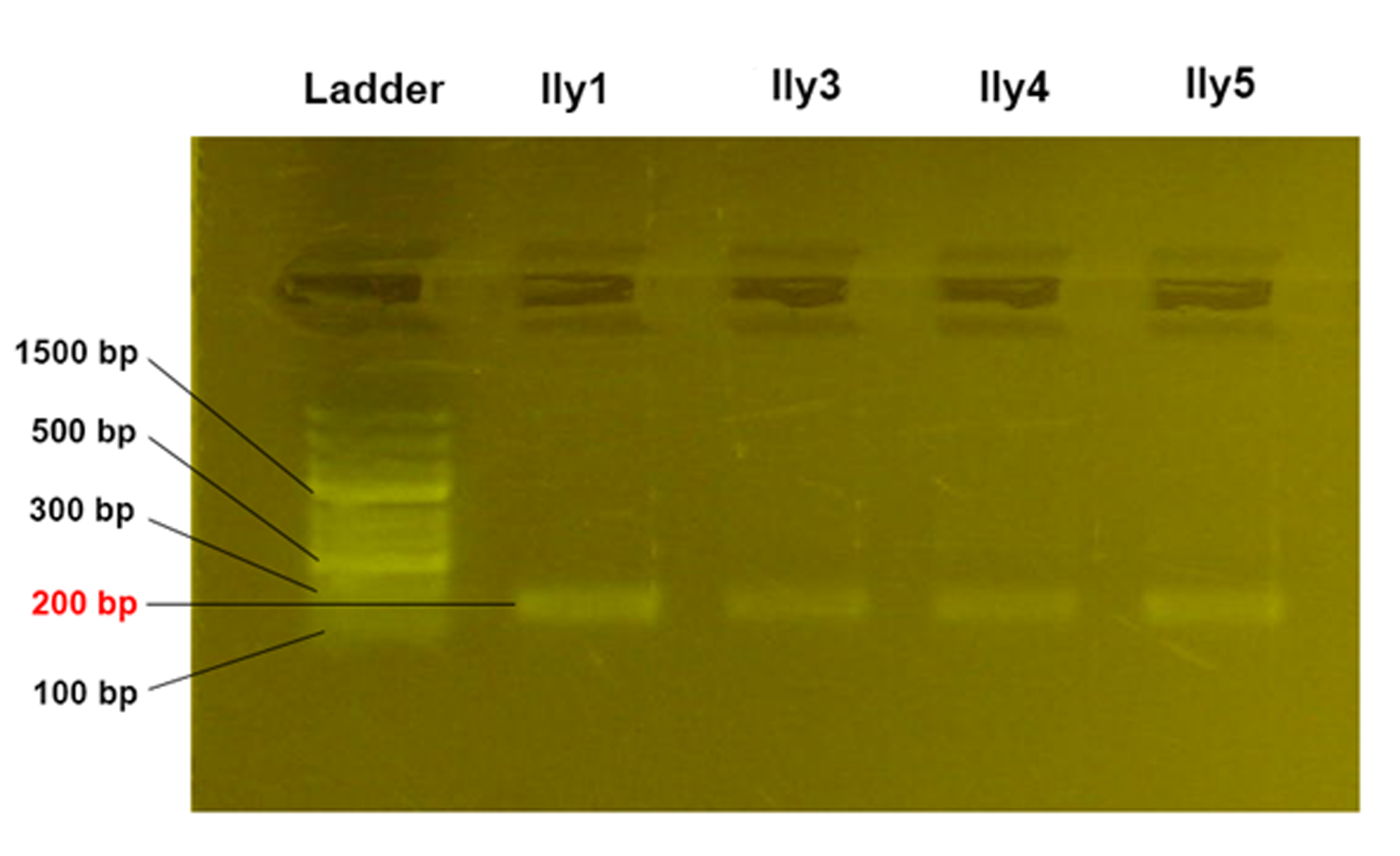

Supplement: S1 Fig — A clearly detectable amplicon at the expected size (~200 dp) in case of all four tested fungal isolates (Ily1, Ily3, Ily4, Ily5) was observed. (TIF) [file pone.0339616.s001.tif]

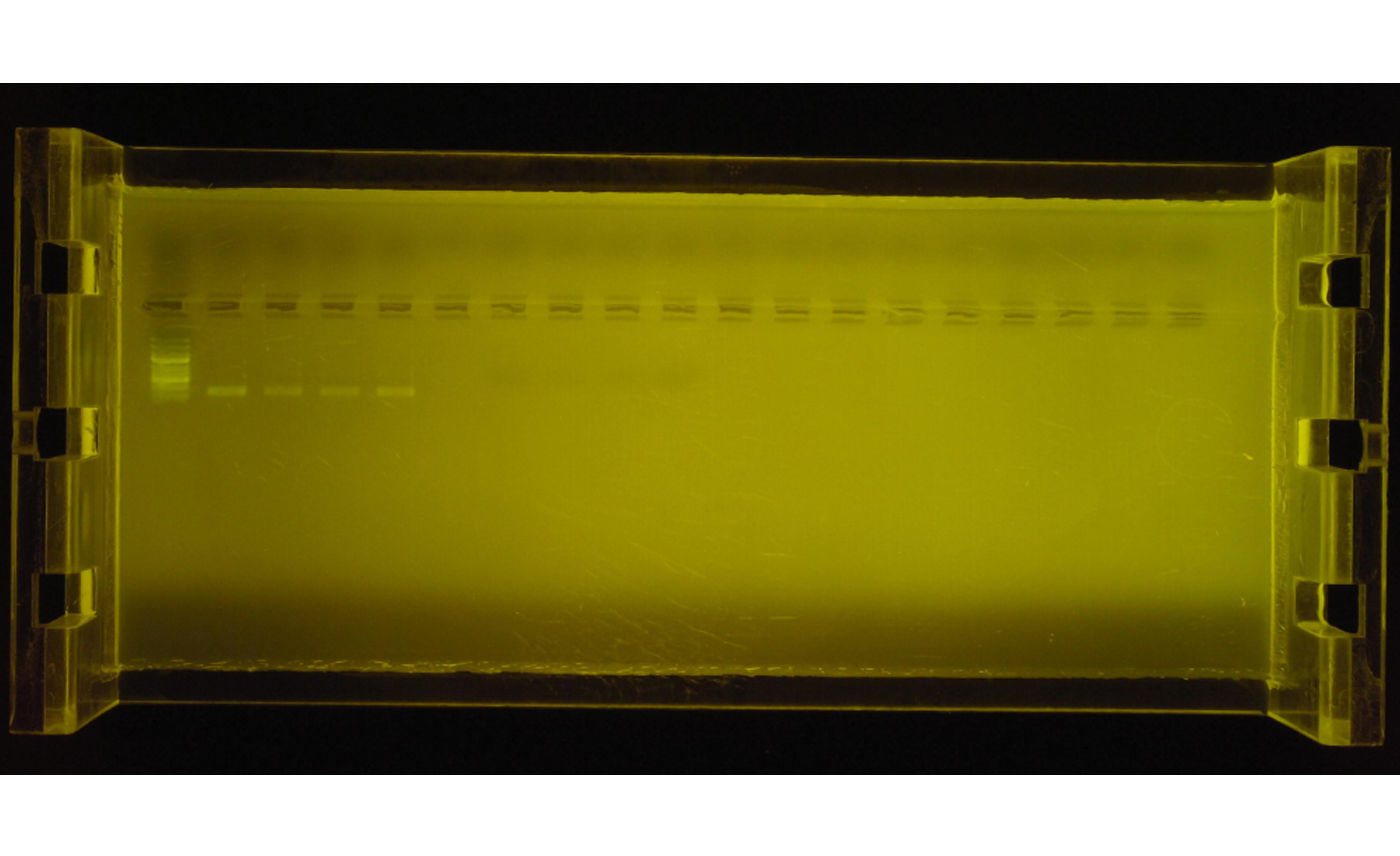

Supplement: S2 Fig — (TIF) [file pone.0339616.s002.tif]
