# Supplementary material for: Isolation and characterization of the phytopathogenic fungus Ilyonectria liriodendri from persimmon as a new susceptible host
Source: PLoS One. 2025 Dec 26;20(12):e0339616. doi: 10.1371/journal.pone.0339616 (PMC12742776; doi:10.1371/journal.pone.0339616)
Supplement: Attachment — Submitted filename: Response to Reviewer2.docx [file pone.0339616.s006.docx]

**Response to Reviewer #2**

*We would like to thank Reviewer#2’s important comments and suggestions. We think that the manuscript has significantly been improved with the implementation of the recommended changes. Please find below our responses to Reviewer#2’s comments (in bold).*

**Reviewer#2 comments:
1- The abstract presents a clear and scientifically sound summary of the study, demonstrating proper application of molecular identification and pathogenicity testing according to Koch’s postulates. However, to enhance its scientific precision, consider refining the structure and clarity of certain parts. The background could briefly emphasize the economic or ecological relevance of Ilyonectria infections.**

*We could not find specific literature data on the economic and ecological significance of black foot diseases. Some references were added to the text with information on disease and pathogen high incidence worldwide.*

**Additionally, when mentioning the phylogenetic analysis, specify the reference strains or databases used for comparison.**

*The resources used in the phylogenetic analysis are listed in Table1. Links added to the resource webpages in the text.*

**Finally, the conclusion effectively highlights the novelty of the finding, but it could be strengthened by indicating its potential implications for disease management in Diospyros virginiana.**

*Thank you for the suggestion! Some recommendations for management techniques against black foot disease pathogens in persimmon are added to the conclusion section.*

**2- The introduction successfully builds the context for identifying Ilyonectria liriodendri as a new pathogen on persimmon. However, a few small improvements could make it stronger. The text could flow more smoothly by shortening long sentences and combining related ideas.**

*The introduction was rewritten in the manuscript according to the above suggestion.*

**The significance of finding I. liriodendri on Diospyros virginiana could be emphasized more clearly as a new contribution to plant pathology. Also, ensure that all scientific names are consistently.**

*Thank you for the suggestion! We tried to emphasize this finding more clearly in the revised manuscript. Scientific names were checked (some typos in the original manuscript were due to autocorrection).*

**3-Material s and Methods
a. Isolation of fungal strains
Please provide more details about the number of samples collected, the conditions of incubation (duration and light/dark), and whether sterilization controls were used. This information will improve the clarity and reproducibility of the isolation method.**

*Suggested information was added to the revised text. No sterilization control was used. However, this sterilization method is widely used and can be considered reliable. In the case of very thin plant samples even no fungal growth occasionally occurs.*

**b. The molecular identification and phylogenetic analysis are appropriate and thorough, but please correct minor grammatical errors (e.g. its suitable → it is suitable) , adjust phrasing for consistency (e.g. Visualization of the phylogenetic tree were done → was performed),**

*Grammar and spelling were checked, and several corrections were made.*

**and briefly explain the need for species-specific PCR in addition to multi-locus sequencing to improve clarity and reproducibility.**

*Suggested information was added to the revised text.*

**c. Check the spelling of the species name (e.g. I. liriodendra → I. liriodendri) and correct the figure references (e.g., diffuse central (a), diffuse lateral (c), or spot-like (c)).**

*Suggested corrections were made.*

**It would also help to clarify why some isolates caused symptoms more frequently than others—whether this is due to differences in virulence, inoculum concentration, or plant susceptibility. Including quantitative measurements of necrosis and noting if statistical analyses were done would make the results stronger.**

*Quantification of necrotic areas was done, but there were no significant differences. We think that our data are not suitable for the comparison of the virulence of strains because of the following reasons: while the number of pathogenicity test replicates are suitable to test Koch’s postulates, but not sufficient to do statistics 3) symptom severity showed high variance even within a single strain 4) it is hard to compare the severity of different types of necrosis (diffuse or point-like).*

**Finally, it would be helpful to clearly explain the difference between natural and experimental infections and why this pathogen on persimmon may have been overlooked before, to make the discussion more informative.**

*The differences between natural and artificial infections are discussed in the revised manuscript. Thank you for the suggestion!*

**d. Try breaking long sentences into shorter ones and separate the ideas about the host being grown outside its native range, the different climate, and exposure to unfamiliar fungal strains. It would also help to clarify the soil DNA study, noting that ITS sequencing can’t always distinguish closely related Ilyonectria species. These small changes would make the discussion more readable and strengthen your argument.**

*The discussion is rewritten according to your suggestion.*

**4- Results and Discussion
1) Language Comments
Line 160–161: Replace (declining D. virginiana plants) with (declining D. virginiana plants) (italicize species name)**

*Suggested correction was made.*

**Line 161–162: The phrase (resulting from their necrosis and/or occlusion) could be rephrased for clarity as (resulting from tissue necrosis and/or vascular occlusion).**

*The sentence was rephrased.*

**Line 167: The phrase (isolated from the symptomatic plants) should read (isolated from symptomatic plants) (remove (the)).**

*Suggested correction was made.*

**Line 168: Replace (while there were slight) with (****although slight differences were observed).**

*Suggested correction was made.*

**Line 169–170: Replace “differences between the isolates(with “differences among the isolates)**

*Suggested correction was made.*

**Line 170–171: Reword description of microconidia, macroconidia, and absence of chlamydospores for readability.**

*The sentence was rewritten according to your suggestion.*

**Line 171–174: Correct grammar and tense in the sentence about gene sequencing and multi-locus phylogenetic analysis.**

*The sentence was rewritten.*

**Line 177–178: Fix spelling (“constracted” → “constructed”) and improve sentence structure about the phylogenetic tree.**

*Suggested correction was made.*

**Line 179–181: Reword PCR result sentence for clarity and consistency.**

*The sentence was rewritten.*

**Line 266: Integrate incomplete sentence (a significant crop [31]) with the previous sentence for clarity.**

*The sentence was rewritten according to your suggestion.*

**Line 267: Reword (growing of the host out of its native range) → (****cultivation of the host outside its native range).**

*Suggested correction was made.*

**Line 268: Italicize species name (D. virginiana) and improve phrasing of climatic exposure.**

*Suggested correction was made.*

**Line 269: Rephrase (encounters fungal strains it has not evolved to coexist with) → (****encounters fungal strains with which it has not co-evolved).**

*The sentence was rewritten.*

**Lines 270–271: Simplify sentence about hidden infections, non-specific symptoms, and multiple pathogens.**

*The sentence was rewritten.*

**Line 272: Remove comma in (It is also notable, that…) → (It is also notable that…).**

*Suggested correction was made.*

**Line 273: Improve flow: (surrounded by numerous vineyards) → (which is surrounded by numerous vineyards).**

*Suggested correction was made.*

**Line 274: Replace (There is no literature data…) → (****No literature reports…).**

*Suggested correction was made.*

**Lines 275–276: Clarify DNA metabarcoding sentence; italicize Ilyonectria.**

*The sentence was rewritten.*

**Lines 277–278: Reword (which is not suitable for distinguishing black foot disease pathogens at the species level) → (****which is not suitable for species-level identification of black foot disease pathogens).**

*The sentence was rewritten.*

**2) Scientific comments
• Fungal Isolation and Morphology ( Line 168):
The phrase (similar colony morphology as described for I. liriodendri [30]) could be supported with microscopic features (e.g., conidial size).**

*Quantitative data on conidial size were added to the revised text.*

**• Micro- and Macroconidia Description (Lines 169–171):
- Providing actual measurements (in μm) for microconidia and macroconidia would strengthen the morphological identification.**

*Conidial dimensions were added to the revised text.*

**- The absence of chlamydospores is mentioned, but noting whether this was consistent across all isolates would be useful.**

*Suggested information is added to the revised text.*

**• Strain Differentiation (Line 190):
The statement that (morphological differences…suggest that each of them represents a different strain ) is reasonable, but it would be strengthened by providing quantitative measurements such as colony diameter, hyphal characteristics, or conidial dimensions.**

*Quantitative data on conidia length were added to the revised text where differences were mentioned.*

**• Introduction to Exoenzyme Analysis (Lines 224–225):
- Consider briefly explaining the rationale for studying exoenzyme production in I. liriodendri (e.g., relevance to pathogenicity, virulence, or ecological adaptation).**

*The suggested information is added to the revised text.*

**• (Line 230) : Mention whether cellulase activity correlates with observed pathogenicity on host tissue.
• (Line 231) : The reduced activity in Ily4 could be discussed in the context of strain variability and potential ecological or pathogenic implications**

*The differences between fungal strains regarding exoenzyme production and pathogenicity were discussed in the revised text. We think that clear correlation (or the lack of correlation) between enzyme activity and virulence should not be stated according to our experiments since: 1) enzyme activity values can differ in vitro and in planta 2) the number of pathogenicity test replicates are suitable to test Koch’s postulates, but not sufficient to do statistics 3) symptom severity showed high variance even within a single strain 4) it is hard to compare the severity of different types of necrosis (diffuse or point-like). Some of the above thoughts are included in the revised manuscript.*

**• (Line 279): Discuss the ecological and epidemiological implications of latent soil populations serving as a local source for disease, including potential spread to nearby susceptible hosts.**

*Information on the spreading of black foot disease pathogens was added to the revised text.*

**5- Conclusion
• Lines (283–284):
- The statement (I. liriodendri contributes to the development of vascular fungal infection in a persimmon species, D. virginiana) is clear, but consider emphasizing whether this conclusion is based on morphological, molecular, or pathogenicity evidence.
- You could also clarify that this is the first report of I. liriodendri infecting D. virginiana, if applicable.**

*The suggested information was added to the revised manuscript.*
